# Development of a Method to Monitor Gene Expression in Single Bacterial Cells During the Interaction With Plants and Use to Study the Expression of the Type III Secretion System in Single Cells of *Dickeya dadantii* in Potato

**DOI:** 10.3389/fmicb.2018.01429

**Published:** 2018-06-28

**Authors:** Zhouqi Cui, Xiaochen Yuan, Ching-Hong Yang, Regan B. Huntley, Weimin Sun, Jie Wang, George W. Sundin, Quan Zeng

**Affiliations:** ^1^Department of Plant Pathology and Ecology, The Connecticut Agricultural Experiment Station, New Haven, CT, United States; ^2^Department of Biological Sciences, University of Wisconsin-Milwaukee, Milwaukee, WI, United States; ^3^Guangdong Key Laboratory of Integrated Agro-environmental Pollution Control and Management, Guangdong Institute of Eco-environmental Science and Technology, Guangzhou, China; ^4^Department of Plant Biology, Michigan State University, East Lansing, MI, United States; ^5^Department of Plant, Soil, and Microbial Sciences, Michigan State University, East Lansing, MI, United States

**Keywords:** *Dickeya dadantii*, T3SS, bacterial virulence, single cell techniques, soft rot

## Abstract

*Dickeya dadantii* is a bacterial plant pathogen that causes soft rot disease on a wide range of host plants. The type III secretion system (T3SS) is an important virulence factor in *D. dadantii*. Expression of the T3SS is induced in the plant apoplast or in *hrp*-inducing minimal medium (hrp-MM), and is repressed in nutrient-rich media. Despite the understanding of induction conditions, how individual cells in a clonal bacterial population respond to these conditions and modulate T3SS expression is not well understood. In our previous study, we reported that in a clonal population, only a small proportion of bacteria highly expressed T3SS genes while the majority of the population did not express T3SS genes under hrp-MM condition. In this study, we developed a method that enabled *in situ* observation and quantification of gene expression in single bacterial cells *in planta*. Using this technique, we observed that the expression of the T3SS genes *hrpA* and *hrpN* is restricted to a small proportion of *D. dadantii* cells during the infection of potato. We also report that the expression of T3SS genes is higher at early stages of infection compared to later stages. This expression modulation is achieved through adjusting the ratio of T3SS ^ON^ and T3SS ^OFF^ cells and the expression intensity of T3SS ^ON^ cells. Our findings not only shed light into how bacteria use a bi-stable gene expression manner to modulate an important virulence factor, but also provide a useful tool to study gene expression in individual bacterial cells *in planta*.

## Introduction

*Dickeya dadantii* is a Gram-negative plant pathogenic bacterium that causes soft-rot, stem wilt, and blight diseases on a wide range of economically important crops including potato, carrots, and cabbage. Infection caused by *D. dadantii* can occur in multiple organs such as tubers, stems, leaves, and flowers, causing localized symptoms (Nelson, 2009). Under favorable conditions such as high temperature (35°C) and humidity (100% relative humidity), the pathogen can further spread through the xylem tissues and cause systemic wilt symptoms (Nelson, 2009; Tsror et al., 2009). In addition to the damage in the field, infections caused by *D. dadantii* can also lead to economically significant losses in postharvest storage (Pérombelon, 2002).

Multiple virulence factors of *D. dadantii* are important for the successful infection of plant hosts. Among them, the type III secretion system (T3SS), a needle-like structure that translocates effector proteins into the plant cytosol, is required for the full virulence of *D. dadantii* (Yang et al., 2002; Charkowski et al., 2012). The T3SS in *D. dadantii* is encoded by a cluster of 35 *hrp, hrc*, and *dsp* genes. These genes encode the structural components of the T3SS apparatus, as well as harpin proteins, translocators, and effectors. Although the exact consequence of the translocation of T3 effectors of *D. dadantii* into host plants is still not clear, it is believed that at least one effector, DspE, may induce plant cell death and promote maceration of plant tissue, similar to other soft rot bacteria (Kim et al., 2011; Charkowski et al., 2012). As a necrotroph, *D. dadantii* harbors fewer T3 effectors than other hemi-biotrophic pathogens such as *Pseudomonas syringae*.

The expression of T3SS genes is induced under *hrp-*inducing conditions. For most phytopathogenic bacterium, the *hrp-*inducing conditions are nutrient-deficient, low osmolyte, low Ca^2+^, and slight acidic (pH = 5.5), such as in leaf apoplast or in *hrp-*inducing minimal media (hrp-MM; Rahme et al., 1992; Schulte and Bonas, 1992; Francis et al., 2002; Brencic and Winans, 2005; Yahr and Wolfgang, 2006), and repressed under nutrient-rich conditions such as Luria–Bertani (LB) medium (Tang et al., 2006). As the pathogens often interact with different host organs with various nutrient contents and immunity, the expression of the T3SS is tightly controlled by a complex network composed of regulators such as alternative sigma factors, two-component signal transduction systems, proteases, and regulatory small RNAs (Tang et al., 2006; Yang et al., 2008b; Li et al., 2010; Zeng et al., 2010, 2013; Zeng and Sundin, 2014).

Although the induction signals for T3SS expression were characterized and some genetic regulators of T3SS were identified, how individual cells in a bacterial population respond to environmental signals and turn on or off T3SS expression is still not well understood. As bacteria are single-cell microorganisms and multiply through asexual reproduction, it is easy to assume that all cells in a genetically homogenous population may uniformly respond to environmental signals, and display similar levels of gene expression. However, using single cell technology, our previous study demonstrated that the expression of T3SS genes in a bacterial population is heterogeneous under *in vitro* conditions: multiple T3SS genes including *hrpA* (encoding the *hrp* pilus), *hrpN* (encoding a harpin protein), and *dspE* (encoding a T3 effector) are highly expressed in only a small proportion of the cells, and are not expressed in the rest of the *D. dadantii* population under the *hrp-*inducing conditions (hrp-MM; Zeng et al., 2012). This phenotype of two sub-populations displaying different levels of gene expression in a homogeneous bacterial population was defined as bi-stability (Zeng et al., 2012). Although the expression of T3SS genes in *D. dadantii* is bi-stable under *in vitro* induction conditions, whether the expression is bi-stable in the plant apoplast during the actual host–pathogen interaction is still unknown.

Compared to a homogenous liquid culture of hrp-MM, the plant apoplast is much more complex, containing not only different structures (e.g., stomata, mesophyll tissue, and xylem tissue), but also plant cell debris as a result of infection. Thus, it is much more difficult to visualize the bacteria from an infected plant than from a liquid pure culture. Staining bacterial cells with fluorescent dye can help localize bacteria cells in plants (Rufian et al., 2016); however, as bacteria are often embedded in the plant tissues and may not be easily reached by the staining chemicals, the staining and visualization steps were performed after releasing the bacteria from the plant tissue (Rufian et al., 2016). Several reporter systems have been developed for the visualization and monitoring gene expression, such as the single-cell bioreporters of GFP (Leveau and Lindow, 2001a,b; Remus-Emsermann and Leveau, 2010), and GFP with mRFP (Dulla and Lindow, 2008), and most of the work is done in hemi-biotrophic pathogen *P. syringae* or *Pantoea agglomerans* (formerly named *Erwinia herbicola*) on a phylosphere environment (Leveau and Lindow, 2001a,b; Dulla and Lindow, 2008; Remus-Emsermann and Leveau, 2010). So far there is no established protocol of visualizing and quantifying the expression of a target gene in single cells of necrotrophic plant pathogen during their infection of plant hosts.

In this study, we developed a dual fluorescence reporter system and successfully used it in characterizing the bi-stable expression of T3SS in *D. dadantii* during its interaction with potato (*Solanum tuberosum*). The dual reporter we used enables the visualization of the total bacterial population using green fluorescence while monitoring the expression of T3SS genes in single cells with red fluorescence. Using this method, we demonstrated the expression dynamics of T3SS genes in single cells of *D. dadantii* during the early and late stages of infection in various plant tissue types. Our results suggest that by modulating the percentage of the T3SS^ON^ cells in the total population and the expression intensity of the T3SS^ON^ cells, *D. dadantii* maintains a higher overall expression of T3SS at the early stages of infection than at the late stage of infection in potato.

## Materials and Methods

### Bacterial Strains and Growth Conditions

Bacterial strains, plasmids, and primers used in this study are listed in **Table 1**. *D. dadantii* 3937 was stored at –80°C in 15% glycerol and grown in LB medium at 28°C. *Escherichia coli* was cultured in LB medium at 37°C. The antibiotic ampicillin (Ap) was supplemented to the media at a final concentration of 100 μg/ml.

**Table 1:** Strains, plasmids, and primers used in this study.

Strains, plasmids, and primers	Characters or sequences (5’ to 3’)^a^	Reference or source
**Strains**		
*Escherichia coli*	DH5α	
*Dickeya dadantii*	Wild type strain 3937, isolated from *Saintpaulia* (African violet)	Yang et al., 2002
*D. dadantii* 3937 (pP*nptII*-P*hrpA*)	*D. dadantii* containing dual-fluorescence promoter reporter plasmid *nptII*-*gfp*-*hrpA*-*mCherry*, Ap^R^	This study
*D. dadantii* 3937 (pP*nptII*-P*hrpN*)	*D. dadantii* containing dual-fluorescence promoter reporter plasmid *nptII*-*gfp*-*hrpN*-*mCherry*, Ap^R^	This study
*D. dadantii* 3937(pP*nptII*-P*pelD*)	*D. dadantii* containing dual-fluorescence promoter reporter plasmid *nptII*-*gfp*-*pelD*-*mCherry*, Ap^R^	This study
**Plasmids**		
pPROBE-AT	Promoter-probe vector, Ap^R^	Miller et al., 2000
*nptII*-*gfp*-*hrpA*-*mCherry*	pPROBE-AT with transcriptional fusions of *nptII* promoter-*gfp* and *hrpA* promoter-*mCherry*, Ap^R^	This study
*nptII*-*gfp*-*hrpN*-*mCherry*	pPROBE-AT with transcriptional fusions of *nptII* promoter-*gfp* and *hrpN* promoter-*mCherry*, Ap^R^	This study
*nptII*-*gfp*-*pelD*-*mCherry*	pPROBE-AT with transcriptional fusions of *nptII* promoter-*gfp* and *pelD* promoter-*mCherry*, Ap^R^	This study
**Primers**		
*nptII* promoter forward	GGTGGATCCACTGGGCTATCTGGACAAGG	This study
*nptII* promoter reverse	GTTGAATTCAATCATGCGAAACGATCCTC	This study
*hrpA* promoter forward	GTTGGATCCTCTACTTCCGGCTGGATACG	This study
*hrpA* promoter reverse	GGTGAGCTCGATAAATATCTCCAGTTAAC	This study
*hrpN* promoter forward	AAGGATCCCCGTCGTCATCAACAGGC	This study
*hrpN* promoter reverse	CATGAGCTCGAGCGACGCACCAAGCTG	This study
*pelD* promoter forward	GTTGGATCCCGATGTTCTCCGAAG	This study
*pelD* promoter reverse	CTTGAGCTCAGACTGGTTCCTTGC	This study
*mCherry* forward	AAAGAGCTCAAGGAGGAAAAACATATGGTGAGCAAGGGCGAGGA	This study
*mCherry* reverse	TCCGTCGACTTACTTGTACAGCTCGTCCA	This study
*hrpA* qPCR forward	CAGCAATGGCAGGCATGCAG	Zeng et al., 2010
*hrpA* qPCR reverse	CTGGCCGTCGGTGATTGAGC	Zeng et al., 2010
*hrpN* qPCR forward	TCGGCAGCGGTCTGAACGAC	Zeng et al., 2010
*hrpN* qPCR reverse	CCAGCGACAACGGCGAGAA	Zeng et al., 2010
*dspE* qPCR forward	GATGGCGGAGCTGAAATCGTTC	Zeng et al., 2010
*dspE* qPCR reverse	CCTTGCCGGACCGCTTATCATT	Zeng et al., 2010
*rplU* qPCR forward	GCGGCAAAATCAAGGCTGAAGTCG	Zeng et al., 2010
*rplU* qPCR reverse	CGGTGGCCAGCCTGCTTACGGTAG	Zeng et al., 2010

*^*a*^Ap^*R*^, ampicillin resistance; underlined sequences, restriction enzyme sites; *gfp*, green fluorescent protein gene; *mCherry*, red fluorescent reporter protein gene.*

### Construction of the Dual Fluorescence Reporter Plasmids

We first constructed the *nptII-gfp-hrpA-mCherry* plasmid using pPROBE-AT as the backbone. The promoter of *hrpA*, promoter of *nptII*, and *mCherry* gene were PCR amplified using the chromosomal DNA of *D. dadantii* 3937, pKD4 plasmid (Datsenko and Wanner, 2000), and pMEmCherry plasmid (Kwan et al., 2007) as templates, respectively, using the primers listed in **Table 1**. The three PCR products were purified with a QIAquick PCR purification kit (Qiagen, Valencia, CA, United States), and were digested by restriction enzymes: *hrpA* promoter was digested by *SacI* and *BamHI*; mCherry was digested by *SacI*; and *nptII* promoter was digested by *BamHI*. The three digested fragments were ligated with a 1:1:1 ratio using T4 DNA ligase (Promega, Madison, WI, United States). The *nptII-hrpA-mCherry* fusion construct was acquired by PCR using the ligation product as template and the mCherry reverse and *nptII* promoter reverse as primers (**Table 1**). The PCR fusion construct was gel purified, digested with *EcoRI* and *SalI*, and cloned into the pPROBE-AT between the *EcoRI* and *SalI* sites.

The *nptII-gfp-hrpN-mCherry* and *nptII-gfp-pelD-mCherry* plasmids were subsequently constructed by replacing the *hrpA* promoter of *nptII-gfp-hrpA-mCherry* with the promoters of *hrpN* or *pelD*. Briefly, promoters of *hrpN* and *pelD* were PCR amplified from the *D. dadantii* 3937 chromosome and were digested by *BamHI* and *SacI* enzymes. *nptII-gfp-hrpA-mCherry* plasmid was also digested with BamHI and SacI to release the *hrpA* promoter. The promoters of *hrpN* and *pelD* were ligated to the digested vector resulting in the *nptII-gfp-hrpN-mCherry* and *nptII-gfp-pelD-mCherry* plasmids. High-fidelity Taq DNA polymerase (Platinum Taq from Thermo Fisher, Waltham, MA, United States) was used in all PCR amplifications. All constructs were confirmed by sequencing.

### Inoculation of *D. dadantii* Into Potato

Potato “Russet” (*S. tuberosum* “Russet Burbank”) plants were grown and inoculations were conducted in a growth chamber at 23°C, 95% humidity, with artificial light maintained for 16 h periods within the 24 h cycle. Fifteen-weeks-old potato plants were used for leaf and stem inoculation assay. Potato tubers used in the tuber inoculation assay were sliced into 15 mm slices, placed on a wet filter paper in a Petri dish, and enclosed by the Petri dish lid to maintain high humidity.

The inoculum was prepared as follows. *D. dadantii* 3937 strains carrying different promoter reporter plasmids were grown on LB + Ap plates for 48 h at 28°C. Cells from a single colony were inoculated into LB + Ap broth and were cultured in a shaker incubator overnight. Cells were collected by centrifugation at 4600 *g* for 5 min, and were adjusted to a final concentration of 1 × 10^8^ CFU/ml in 0.5 × phosphate buffer saline (PBS). For leaf inoculation, young potato leaves were infiltrated with the bacterial suspension using a needless syringe (10 ml). For stem inoculation, young potato stems were infiltrated with bacterial suspension using a 10 ml syringe attached with a 0.3 mm × 13 mm needle. Inoculated leaves and stems were bagged with a plastic Ziploc bag to maintain high humidity conditions. For tuber inoculation, 100 μl of bacterial suspension was added on top of the prepared tuber slices in petri dishes. Inoculated potato leaves, stems, and tuber slices were harvested at 12, 24, 48, and 72 h after inoculation and were directly used for symptom development, confocal microscopy, flow cytometry, and quantitative reverse transcription PCR (qRT-PCR). Each experiment contains 5–10 biological replicates and has been repeated at least two times.

### qRT-PCR Analysis

Total RNA was isolated from symptomatic plant tissue using RNeasy Plant Mini Kit (Qiagen, Valencia, CA, United States), and was treated with Turbo DNase (Ambion, Austin, TX, United States) to remove any genomic DNA contamination. cDNA was synthesized using 1 μg of treated total RNA using the iScript cDNA synthesis kit (Bio-Rad, Hercules, CA, United States). qRT-PCR was performed by adding 1 μl of the cDNA as template in a 20 μl reaction of SsoAdvanced universal SYBR Green supermix (Bio-rad, Hercules, CA, United States). Data were collected by a CFX96 Touch Real-Time PCR machine (Bio-rad, Hercules, CA, United States). Relative expression of *hrpA, hrpN*, and *dspE* to the endogenous control *rplU* was calculated by the ΔΔCt method (Livak and Schmittgen, 2001). The relative expression of each of the four biological replicates, as calculated by 2^-ΔCt^, was also displayed in the figure. Statistic analysis was performed using the one-way analysis of variance (ANOVA) model in the “stats” package in R. The same experiment was repeated twice, with similar results observed each time.

### Confocal Microscopy

**FIGURE 1:**
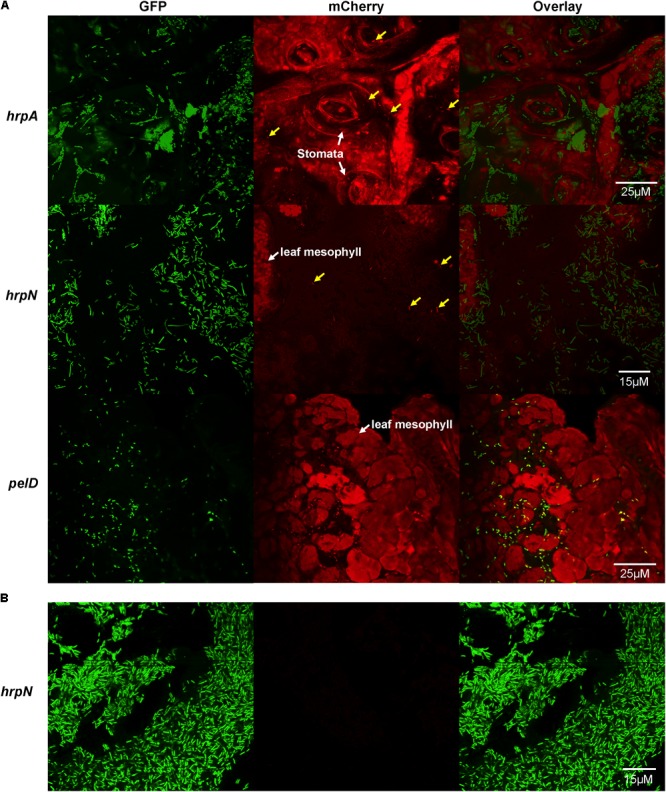
Confocal microscopy observation of T3SS gene expression, as defined by promoter activities of *hrpA, hrpN*, and *pelD* (a non-T3SS control), in single cells of *D. dadantii* in potato leaves. **(A)** Promoter activities of *hrpA, hrpN*, and *pelD* in *D. dadantii* in potato leaves. **(B)** Promoter activity of *hrpN* in LB broth. Potato leaves were infiltrated with *D. dadantii* carrying dual different fluorescence reporter plasmids, *nptII-gfp-hrpA-mCherry, nptII-gfp-hrpN-mCherry*, or *nptII-gfp-pelD-mCherry*, respectively. Leaves with water soaking symptoms 24 h after inoculation were dissected and observed under a confocal microscope. *D. dadantii* carrying *nptII-gfp-hrpN-mCherry* plasmid was cultured in LB broth for 18 h prior to observation. Left panel: GFP fluorescence. Middle panel: mCherry fluorescence. Right panel: overlay of GFP and mCherry.

Symptomatic potato leaves were removed from the plant and kept in enclosed plastic bags prior to observation. Leaves were dissected into 5 mm^2^ pieces with a razor blade, and were mounted on slides in 4 μl of double-distilled H_2_O under a 0.17 mm coverslip. Observation was made using either a Leica SP5 confocal microscope (**Figure 1**) or a Zeiss Axioplan 2 fluorescence microscope. The Leica SP5 confocal microscope (Leica, Wetzlar, Germany) was equipped with four lasers, 405 nm, multi-line Argon, 561 nM and 633 nm, and two HyD detectors. The Zeiss Axioplan 2 fluorescence microscope (Oberkochen, Germany) was equipped fluorescence filter sets 02 EX G365; 10 EX BP450-490,SB; and 15 EX BP 546/12. Deconvolution of confocal microscopic images was performed using Huygens STED deconvolution software (Scientific Volume Imaging, Hilversum, Netherlands). Overlay of green and red fluorescence images was performed using Leica LAS-X software. Each experiment included five leaves as biological repeats and the experiment was repeated three times with similar results observed each time.

### Flow Cytometry and Cell Sorting

Plant tissues with typical soft rot symptoms including water soaking, lesions, and maceration were dissected from the healthy nearby tissues and were placed in a 1.5 ml microcentrifuge tube with 200 μl of 0.5 × PBS. Tissues were macerated with a 1000 μl pipette tip and vortexed for 10 s to release bacterial cells into the suspension. The suspension containing bacterial cells and plant debris was filtered through a 70 μm laboratory sifting mesh (Carolina Biological Supply Company, Burlington, NC, United States) to remove large plant debris. The flow through was diluted 20 times and analyzed by a BD FACS Aria II, equipped with the following lasers and detectors: 100 mW 355 nm UV laser with two PMTs, 200 mW 405 nm violet laser with six PMTs, 200 mW 488 nm blue laser with three PMTs, 200 mW 532 nm green laser with four PMTs, and 150 mW 637 nm red laser with three PMTs. Sample collection and analysis processes were performed in a timely manner to avoid potential alteration of gene expression as a result of maintaining bacterial cells in the PBS buffer. The whole process (from releasing bacteria into PBS to FACS analysis) was performed within 3 min. Five biological repeats were included in each sample at each timepoint. The experiment was repeated three times with similar results observed in each time.

For the cell sorting and re-inoculation experiment, potato leaves were infected with *D. dadantii* carrying *nptII-gfp-hrpN-mCherry* and processed after 18 h. A total of 50,000 GFP positive, mCherry positive and 50,000 GFP positive, mCherry negative cells were collected into two individual 1.5 microcentrifuge tubes, respectively, and were plated on two LB agar plates supplemented with Ap. Ten single colonies from each plate were re-inoculated into LB broth, and subsequently inoculated into new potato leaves. The infected leaves were observed by fluorescence microscope 18 h after inoculation. The experiment was repeated twice with similar results observed in each time.

## Results

### Expression of *hrpA* and *hrpN* Is Bi-stable in *D. dadantii* During the Interaction With Potato

As we observed a bi-stable expression pattern of *hrpA* and *hrpN* in *D. dadantii* 3937 when cultured an *in vitro* condition that mimics the leaf apoplast (hrp-MM) in our previous study (Zeng et al., 2012), we next determined if the expression of the *hrp* genes is indeed bi-stable during the interaction with plant hosts. A dual fluorescence reporter system was constructed to monitor the expression of *hrpA* or *hrpN* in single cells of *D. dadantii*. The dual reporter used in this study contains two fluorescence reporter genes: *gfp* and *mCherry*, which encode green and red fluorescence, respectively (Supplementary Figure S1). We cloned a constitutively expressing promoter, P*nptII*, upstream of *gfp*, and the promoter of interest (*hrpA* or *hrpN*) upstream of *mCherry.* The *nptII-gfp* and *hrpA*/*hrpN*-*mCherry* transcriptional fusions are oriented in opposite directions, and both contain downstream terminator sequences (Supplementary Figure S1).

We examined potato leaves infected with *D. dadantii* carrying the dual fluorescence reporter using fluorescence microscopy. At 24 h post-inoculation, *D. dadantii* cells were green fluorescent and could be easily distinguished from the plant background which is red auto-fluorescent (**Figure 1A**). Out of all green bacteria cells, only a small proportion is red fluorescent, suggesting that both *hrpA* (**Figure 1A**, top) and *hrpN* (**Figure 1A**, middle) are expressed in a bi-stable manner: a small proportion of the population highly expresses these T3SS genes (indicated by arrows) whereas the rest of the population does not express these genes. The T3SS^ON^ cells seem to be evenly distributed and mixed with the T3SS^OFF^ cells, and none of the cell types displays any association with specific plant structure [e.g., stomata, mesosphyll tissue (**Figure 1**)]. Compared to the T3SS genes, the expression of *pelD*, a virulence-related but non-T3SS gene (encoding a pectate lyase), is not expressed in the bi-stable manner (**Figure 1A**, bottom). The expression of *pelD* is similar in all single cells of the *D. dadantii* population. Furthermore, the expression of *hrpN* was not detected in LB broth, a nutrient-rich, *hrp-*repressing condition (**Figure 1B**). Overall, the results suggest that the expression of T3SS genes, such as *hrpA* and *hrpN*, is restricted to a small proportion of *D. dadantii* cells during the infection of potato leaves.

### The Expression of T3SS Is Higher at the Early Stage of an Infection Process Than at Later Stage, and Is Higher in Tuber and Stem Than in Leaf

Next, we investigated the bi-stable expression of T3SS in *D. dadantii* in the context of disease progression in potato. *D. dadantii* encounters different levels of stress and host immunity as the disease infection progresses from the early to late stage of infection and thus may require different levels of T3SS. Additionally, *D. dadantii* may infect different plant organs, which contain various levels of nutrients, pH, osmolality, and immunity. We hypothesize that to adjust to the environmental changes of infection stages and sites, *D. dadantii* may display a dynamic response in the T3SS expression level, which can be reflected at the single cell level.

**FIGURE 2:**
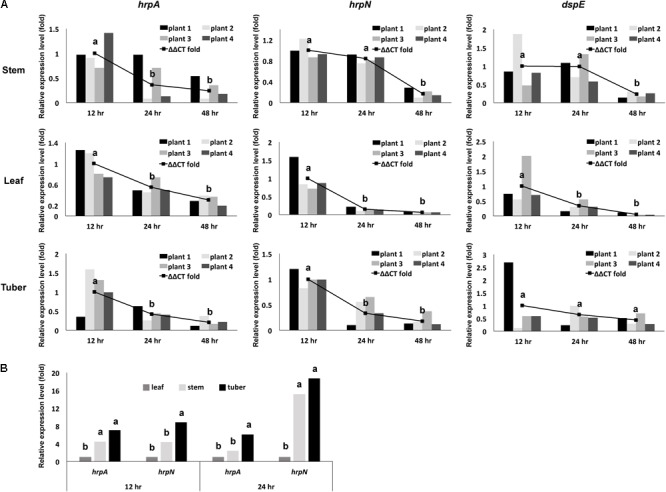
Overall expression of representing T3SS genes in a *D. dadantii* population, as defined by their mRNA level measured by qRT-PCR, at different stages of infection in different potato organs. **(A)** mRNA levels of *hrpA, hrpN*, and *dspE* in *D. dadantii* in potato stem, leaf, and tuber at different infection stages. **(B)** Comparison of mRNA levels of *hrpA* and *hrpN* between different organ types at 12 and 24 h post-incubation. Different potato organs were inoculated with wild type *D. dadantii*. Symptomatic samples were collected at 12, 24, and 48 h post-inoculation for RNA isolation. The abundance of *hrpA, hrpN, dspE*, and *rplU* mRNA was measured by qRT-PCR. Relative expression of *hrpA, hrpN*, and *dspE* to the endogenous control *rplU* was calculated by the ΔΔCt method. The relative expression of each of the four biological replicates, as calculated by 2^-ΔCt^, is also displayed. Statistic analysis was performed using the one-way analysis of variance (ANOVA) model in the “stats” package in R. The presence of different letters indicates significant difference at *P* < 0.05.

First, we determined the overall mRNA levels of T3SS genes in the total bacterial population of *D. dadantii* in different tissue types and infection stages (leaf, stem, and tuber of potato, at 12, 24, and 48 h post-inoculation). Using qRT-PCR, the expression levels of three T3SS genes, *hrpA, hrpN*, and *dspE*, relative to the expression level of a house-keeping gene, *rplU*, were determined. Compared to the expression level at 12 h post-inoculation, a decrease in relative mRNA abundance of *hrpA, hrpN*, and *dspE* was observed at 24 and 48 h post-inoculation (**Figure 2A**). This decrease of T3SS expression at late stage of infection was observed in all three tissue types tested. Additionally, we observed that the expression of *hrpA* and *hrpN* is higher in potato tuber and stem than in potato leaf (**Figure 2B**). When compared with the *in vitro* culture conditions, the expression of *hrpA* and *hrpN* in stem and tuber is similar to their expression in hrp-MM (8 h post-induction). Although the expression of these genes in the potato leaf is significantly lower than in the hrp-MM, it is still about fourfold higher than the expression in LB (data not shown). Thus, these results demonstrate that *D. dadantii* displayed dynamic levels of T3SS expression during the interaction with potato at different infection stages and in different plant organs.

### Modulation of T3SS Expression in the Overall *D. dadantii* Population Is Achieved by Adjusting Both the Percentage of T3SS^ON^ Cells and the Gene Expression Intensity in the T3SS^ON^ Population

To further understand what occurred at the single-cell level that resulted in the change in overall expression of T3SS in the total bacterial population as determined by qRT-PCR, we used the dual fluorescence reporter *nptII-gfp*-*hrpN*-*mCherry* to monitor the transcription of *hrpN* during disease progression in single cells of *D. dadantii*. Using flow cytometry, the GFP and mCherry fluorescence was measured in potato leaf, stem and tuber infected with *D. dadantii* (*nptII-gfp*-*hrpA*-*mCherry*) at 12, 24, 48, and 72 h.

**FIGURE 3:**
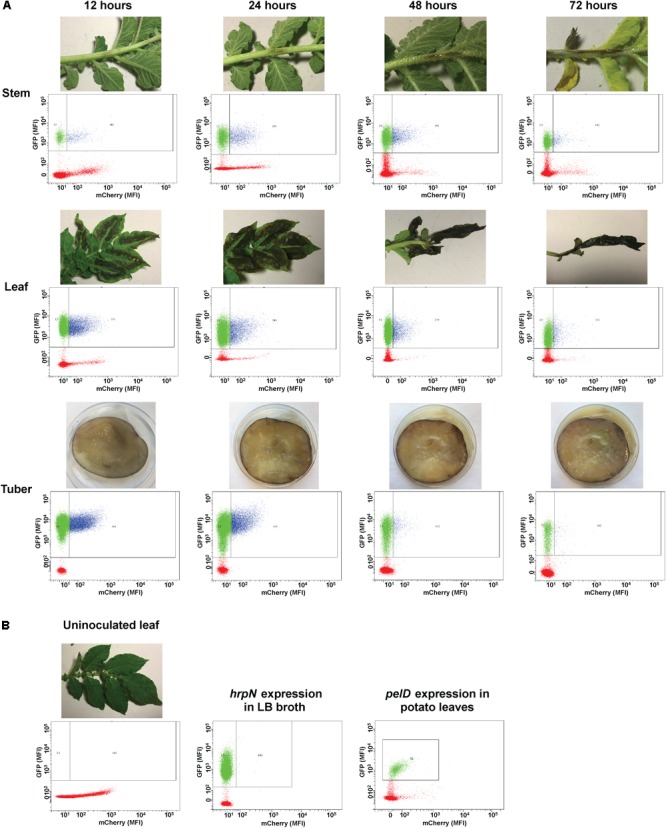
Flow cytometry examination of *hrpN* expression in *D. dadantii* at different stages of infection in potato stem, leaf, and tuber. **(A)** Potato stem, leaf, and tuber infected with *D. dadantii* (*nptII-gfp-hrpN-mCherry*), examined at 12, 24, 48, and 72 h post-inoculation by flow cytometry. Disease symptoms (top) and matrix of red florescence (*x*-axis) – green fluorescence (*y*-axis) determined by flow cytometry (bottom) are displayed. Red dots: plant debris that are GFP negative. Green dots: GFP positive bacteria with low level of *hrpN* expression (mCherry negative). Blue dots: GFP positive bacteria with high level of *hrpN* expression (mCherry positive). **(B)** Flow cytometry measurement of green and red fluorescence of un-inoculated potato leaves, a LB culture of *D. dadantii* (*nptII-gfp-hrpN-mCherry*), and potato leaves infected by *D. dadantii* (*nptII-gfp-pelD-mCherry*).

In non-inoculated plant tissues containing only plant debris particles, no GFP activity was detected (red dots in **Figure 3**). In plant tissues inoculated with *D. dadantii* carrying the dual fluorescence reporter, GFP positive bacteria (blue and green dots in **Figure 3**) can be easily distinguished from the GFP negative plant debris (red dots in **Figure 3**). Additionally, the GFP positive bacterial population was further segregated into two sub-populations based on the segregated expression levels of *hrpN* (represented by the mCherry intensity). A sub-population of *D. dadantii* expressed high level of *hrpN* (mCherry positive, blue dots in **Figure 3A**) whereas the other sub-population expressed low level of *hrpN* (mCherry negative, green dots in **Figure 3A**). The bi-stable expression of *hrpN* in *D. dadantii* was observed in all tissue types tested (stem, leaf, and tuber). Under a T3SS repressive condition (LB broth), no expression of *hrpN* was observed (**Figure 3B**). The expression of a non-T3SS gene, *pelD*, in single cells of *D. dadantii* was also examined, which showed a mono-stable expression pattern in potato leaves (**Figure 3B**). These results demonstrated that the dual fluorescence reporter can be used together with flow cytometry in distinguishing single bacterial cells from the plant debris and quantifying expression of genes of interest in these single bacterial cells.

**FIGURE 4:**
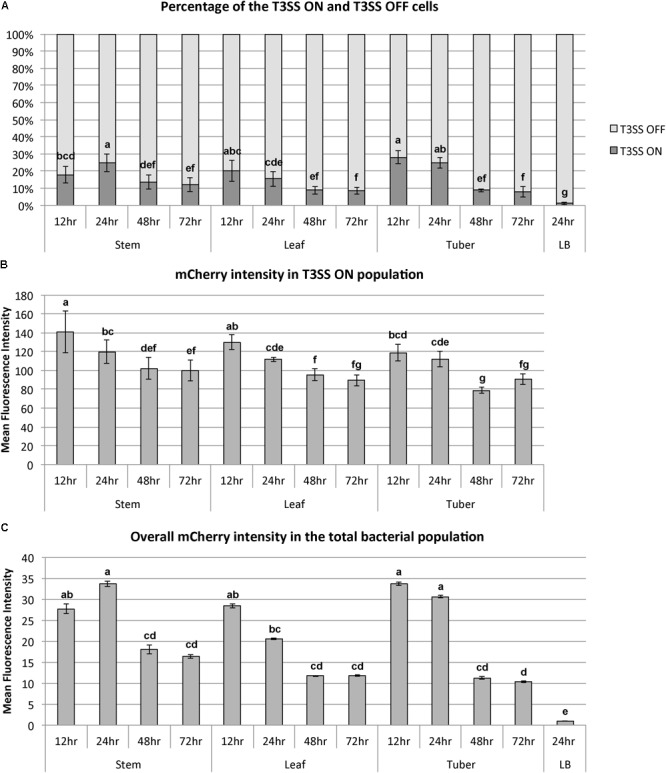
Dynamics of bi-stable expression of *hrpN* in *D. dadantii* during the disease progression in stem, leaf, and tuber of potato. *D. dadantii* carrying the dual fluorescence reporter *nptII-gfp-hrpN-mCherry* was inoculated into stem, leaf, and tuber. Symptomatic tissues were collected at 12, 24, 48, and 72 h post inoculation and were examined by flow cytometry. **(A)** Percentage of cells that highly express *hrpN* (T3SS ^ON^ cells) and do not express *hrpN* (T3SS ^OFF^ cells), as defined by gates used in **Figure 3**. **(B)** Expression level of *hrpN* in the T3SS ^ON^ cells, indicated by the fluorescence intensity of mCherry. **(C)** Overall expression level of *hrpN* in the total population, indicated by the fluorescence intensity of mCherry. Five biological replicates were included. Statistic analysis was performed using the one-way analysis of variance (ANOVA) model in the “stats” package in R. The presence of different letters indicates significant difference at *P* < 0.05.

In a bi-stable gene expression model, the overall expression level of a target gene in the total population is determined by: (1) the percentage of the ON cells in the population and (2) the expression level of the target gene in the ON population. Both the percentage of T3SS ^ON^ cells and the expression intensity in the T3SS ^ON^ cells can be quantified using flow cytometry. During the disease progression from 12 to 72 h, we observed that both the percentage and expression level of the *hrpN* ON cells were reduced. At the early stage (12 h post-inoculation), the *hrpN* ^ON^ *D. dadantii* cells (blue dots in **Figure 3A**) represent about 18–28% of the population in all tissue types (**Figure 4A**). As the infection progressed, this percentage dropped to about 9–13% at 48 h and 8–12% at 72 h post-inoculation (**Figure 4A**), resulting in about a 50% reduction (12–72 h). In addition to the reduction in the percentage of the T3SS^ON^ cells, a reduction in the *hrpN* expression level within the T3SS^ON^ population was also observed. The mCherry intensity, representing the *hrpN* expression level in the T3SS^ON^ population in stem, leaf, and tuber, dropped from 140, 130, and 120 mean fluorescence intensity (MFI) at 12 h to 100, 90, and 90 MFI at 72 h, respectively (**Figure 4B**). However, compared to the percentage difference (50% reduction), this change is considered minor (about 0.3-fold reduction). The cumulative effect of reduction in both percentage and expression intensity caused a 1.5-fold overall reduction in *hrpN* expression level from early to late stage of infection (**Figure 4C**).

### The Bi-stable Expression Pattern Can Derive From a Single ON or OFF Cell

**FIGURE 5:**
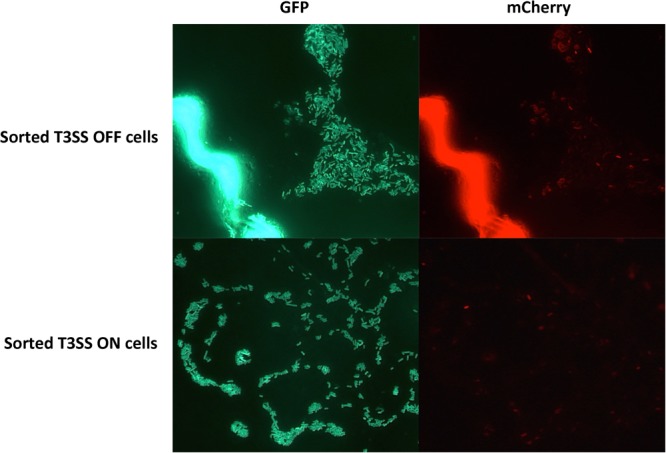
Fluorescence microscopy observation of potato leaves inoculated with cells derived from the T3SS^ON^ and T3SS^OFF^ cells sorted by FACS. *D. dadantii* (*nptII-gfp-hrpN-mCherry*) were inoculated into potato leaves. At 24 h post-inoculation, leaf tissues with water soaking symptoms were dissected and macerated in PBS buffer to release bacterial cells. T3SS^ON^ and T3SS^OFF^ cells were sorted by FACS, recovered on agar plates, and were re-inoculated back into potato leaves. After 24 h, potato leaves inoculated with sorted T3SS ^OFF^ and T3SS ^ON^ cells were examined for green and red fluorescence using microscopy.

Even though we observed that the percentage of T3SS^ON^ cells in the total population varied in different stages of infection, we did not have direct evidence that individual cells in the population can actually switch between the ON and OFF stages. It is possible that the changes in T3SS^ON^ percentage are caused by either an epigenetic mechanism (cells can switch between the two stages), or by a genetic mutation (cells permanently stay in one stage as a result of mutation, percentage changes due to the overgrowth of a sub-population). To test these possibilities, we inoculated potato leaves with *D. dadantii* cells carrying *nptII-gfp*-*hrpA*-*mCherry*. Green fluorescent bacteria were sorted into mCherry positive (T3SS^ON^) and mCherry negative (T3SS^OFF^) populations by a Fluorescence Activated Cell Sorter (FACS). Single *D. dadantii* cells from the sorted populations were recovered on LB agar plates as single colonies. Ten single T3SS^ON^ and 10 single T3SS^OFF^ colonies were sub-cultured in LB broth and inoculated back into potato leaves. We observed in the subsequent populations derived from single colonies of T3SS^ON^ and T3SS^OFF^ cells that both showed bi-stable expression of *hrpN* (**Figure 5**). This observation confirmed that a single cell from either T3SS^ON^ or T3SS^OFF^ populations has the potential to restore bi-stability in the population derived from that single cell. This also suggests that the mechanism governing the T3SS bi-stability is probably an epigenetic mechanism rather than a permanent genetic mutation.

## Discussion

In this study, we demonstrated the feasibility of using a dual fluorescence reporter plasmid in combination with single cell technologies to observe and quantify gene expression at the single cell level during the host–microbe interaction, and also proved that *D. dadantii* truly displayed heterogeneity in expressing T3SS genes during infection of potato. Prior to this study, research in understanding gene expression in single cells of a plant pathogenic bacterial population, especially during the actual interaction with plant hosts, is less understood. It is easy to assume that bacterial cells in a genetically identical population would behave the same while ignoring the possibility of phenotypic heterogeneity. Research in understanding gene expression has been mostly performed in hemi-biotrophic pathogens such as *P. syringae* and on the phylosphere (Leveau and Lindow, 2001a,b; Dulla and Lindow, 2008; Remus-Emsermann and Leveau, 2010; Rufian et al., 2016). Our results improved our understanding of how single cells within a necrotrophic plant pathogenic bacterial population respond to the environmental signals and modulate the expression of the T3SS, one of the most critical virulence factors. The dual fluorescence reporters developed this study also added on to the existing tools in studying phenotypic heterogeneity and the methodology of combining fluorescence microscopy and flow cytometry in monitoring gene expression using this novel vector could be easily transferred to other bacteria–plant interaction systems.

The dual fluorescence reporter system accomplished the goal of monitoring gene expression in single bacterial cells *in planta*. We chose GFP and mCherry as the fluorescence reporter proteins, because unlike other fluorescence colors (such as YFP), the excitation and emission wavelength of GFP and mCherry share minimum overlap, thus allowing specific monitoring of two different promoter activities. Prior to the experimental design, we examined non-infected potato leaves and observed red auto-fluorescence in plant tissue. This urged us to use GFP to label all bacterial cells so that the green cells could stand out from the red plant background (**Figure 1**). The expression of target genes, represented by red fluorescence (mCherry), could then be examined in the green cells only. Despite these efforts, we still found the auto-fluorescence plant tissue may affect the fluorescence microscopy observation when examining a thicker plant tissue. This could be a limitation of our method. The flow cytometry analysis was not affected by the plant auto-fluorescence as the bacterial cells were mechanically released from the plant tissues prior to the analysis.

Compared to integrating the promoter fusions in the bacterial chromosome, using a plasmid as carrier of these promoter fusions as performed in our study has both advantages and disadvantages. Plasmids are easy to manipulate: the promoter of interest can be easily replaced by restriction enzyme digestion (see Supplementary Figure S1), and the plasmids can be easily transferred between strains. The dual reporter plasmid developed in this study is a derivative of pPROBE-AT, which contains a broad-host-range origin of replication, and is compatible with many bacterial species (Miller et al., 2000). Plasmid copy number effect also makes the fluorescence intensity easier to detect than a single-copied construct on the chromosome. However, as there is no antibiotic pressure to maintain the plasmid reporter in plants, we did observe a small proportion of *D. dadantii* cells (about 10%) start to lose the plasmid 4 days after they were inoculated (data not shown). Thus, this method is especially suitable for monitoring gene expression at earlier stages (<96 h). Despite this, the percentage of the ON cells in the total population should not be affected, as the total bacterial population containing the plasmid is controlled by GFP fluorescence.

The temporal expression of T3SS genes in bacterial pathogens has been studied in a few cases and the expression pattern differed in different species of bacteria. In *Vibrio harveyi* and *Parachlamydia acanthamoebae*, the expression of three T3SS operons increased within the early stages of infection and decreased thereafter (Croxatto et al., 2013; Ruwandeepika et al., 2015). However, in *Ralstonia solanacearum*, active expression of T3SS genes was observed throughout its infection *in planta* (Monteiro et al., 2012). Our results suggested that in necrotrophic plant pathogen *D. dadantii*, T3SS plays a more important role at the initial stage of infection than at the late stage of infection. Causing cell death and tissue maceration at an early stage of infection may be critical for the *D. dadantii* to establish a successful invasion. Cell wall degrading enzymes, such as pectate lyases, are considered as the major virulence factors of *D. dadantii* as a necrotrophic pathogen. It is possible that once the initial infection has been established, *D. dadantii* may rely more on these enzymes to further invade the host at later stages of infection. Furthermore, a high level of T3SS expression was observed in potato tuber, an organ with relatively low immunity, represented by the lack of hypersensitive response and less number of resistance genes compared to leaf (Park et al., 2005; Mayton et al., 2010). This observation further supports previously characterized function of T3SS effector in soft rot bacterium, which is to induce plant cell death but not to suppress host immunity (Kim et al., 2011). Due to the limitation of low cell counts, the T3SS expression prior to 12 h was not quantified.

Previous research has suggested that the T3SS may be induced or repressed by certain plant-derived compounds and conditions (Yang et al., 2008a; Anderson et al., 2014). Our results, which suggest the expression of T3SS *in planta* is higher at early stage than later stage, may contribute to the identification of these compounds through metabolic profile comparison of the early and late stages of infection.

Our findings showed that the bi-stable gene expression enables bacterial pathogens to use a multi-tiered regulation of critical virulence factors: it not only allows the adjustment of the percentage of the T3SS ^ON^ population, but also enables the modulation of the expression intensity in the T3SS ^ON^ population. The bi-stable expression pattern may grant bacterial pathogens advantages at the population level. By letting a small proportion of cells establishing a functional of T3SS while maintaining the majority of population free of the T3SS pilus, this may enable the total bacterial population to use an important virulence factor to promote infection while avoiding being detected by the surveillance of plant immunity. Alternatively, it could also be a way of saving energy, as we observed once the population is established the bacteria turned off the T3SS expression at late stage of infection.

The mechanism governing T3SS bi-stability is still not clear. Previously, it is known that key regulators of *hrp* genes such as *hrpL, hrpS, rsmA*, and *rsmB* did not display obvious bi-stable expression pattern in *D. dadantii* (Zeng et al., 2012). Here, we demonstrated that it is caused by an epigenetic mechanism, in which the alternation of cell behavior is not caused by any changes in DNA sequences. Compared to genetic mutations, an epigenetic mechanism allows the cells to switch between the two stages more rapidly. Currently, we are performing transcriptomic analysis of single cells sorted from the two populations to identify the genetic cause of the T3SS bi-stability.

## Author Contributions

QZ, ZC, C-HY, WS, and GS conceived and designed the experiment. ZC, QZ, and XY performed the experiments and analyses. RH, JW, and WS provided technical support for some experiment procedures and the analyses. ZC and QZ wrote the manuscript. All authors read and approved the manuscript.

## Conflict of Interest Statement

The authors declare that the research was conducted in the absence of any commercial or financial relationships that could be construed as a potential conflict of interest.
