# Supplementary figures and images for: Development of a Method to Monitor Gene Expression in Single Bacterial Cells During the Interaction With Plants and Use to Study the Expression of the Type III Secretion System in Single Cells of Dickeya dadantii in Potato
Source: Front Microbiol. 2018 Jun 28;9:1429. doi: 10.3389/fmicb.2018.01429 (PMC6031750; doi:10.3389/fmicb.2018.01429)

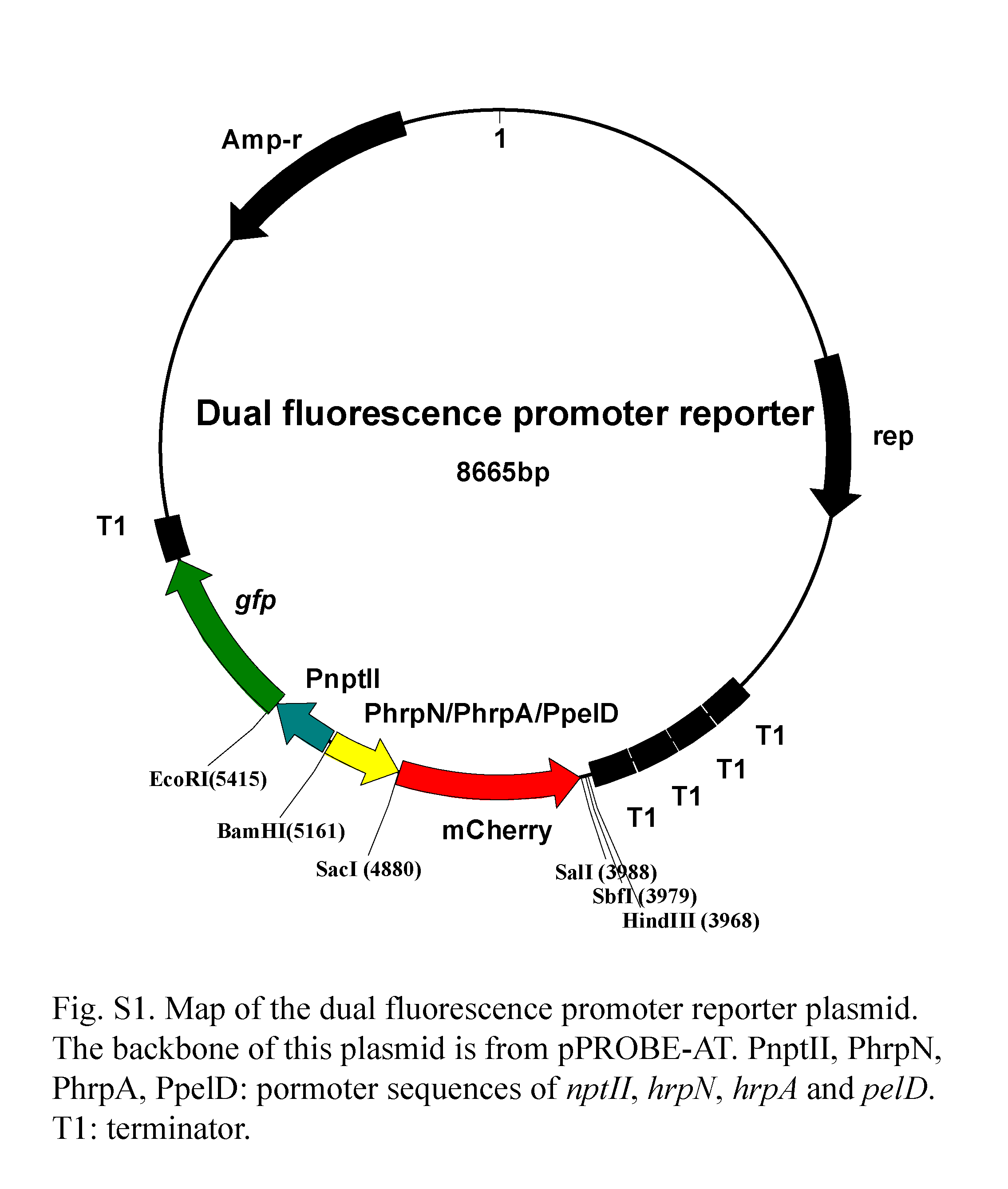

Supplement: Supplementary file 1 [file Image_1.TIF]
